# Salt tolerance mechanisms in Salt Tolerant Grasses (STGs) and their prospects in cereal crop improvement

**DOI:** 10.1186/1999-3110-55-31

**Published:** 2014-03-14

**Authors:** Swarnendu Roy, Usha Chakraborty

**Affiliations:** Plant Biochemistry Laboratory, Department of Botany, University of North Bengal, Siliguri, 734013 West Bengal India

**Keywords:** Salt tolerant grasses (STGs), Osmoprotectants, Reactive oxygen species (ROS), Model STGs, Transgenic plants

## Abstract

Increasing soil salinity in the agricultural fields all over the world is a matter of concern. Salinity poses a serious threat to the normal growth and development of crop plants. What adds to the concern is that all the cereal crops are sensitive to increasing soil salinity. So it is implacable to either search for salinity resistant varieties of crop plants or transform them genetically to sustain growth and reproducibility at increasing salinity stress. For the second perspective, mining the salt tolerant genes in the close relatives of cereal crops apparently becomes important, and most specifically in the salt tolerant grasses (STGs). STGs include the halophytes, facultative halophytes and salt-tolerant glycophytes of the family Poaceae. In this review the potentiality of STGs has been evaluated for increasing the salinity tolerance of cereal crops. STGs are capable of surviving at increasing salt stress by utilizing different mechanisms that include vacuolization of toxic Na^+^ and Cl^-^ in mature or senescing leaves, secretion of excess salts by salt glands, accumulation of osmolytes like proline and glycine betaine, and scavenging of ROS by antioxidative enzymes. The STGs are a therefore a potent source of salt tolerant genes.

**Electronic supplementary material:**

The online version of this article (doi:10.1186/1999-3110-55-31) contains supplementary material, which is available to authorized users.

## Review

### Introduction

Salinity is one of the major abiotic stresses that hinder the performance of the crop plants all over the world. In most crop plants, the main toxic component of salinity is Na^+^ and Cl^-^, which interferes with the normal physiological processes, such as enzyme activities and protein synthesis, as well as causing osmotic imbalances (Munns and Tester 2008). Due to these toxic effects, crops grown on saline soils have significantly reduced yield (Lauchli and Grattan 2007). According to UNEP Report, globally some 20% of agricultural land under irrigation has become salt affected (Nellemann et al*.* 2009). It is known that 40% of the increase in food production over the last 50 years came from irrigated land (Prakash and Stigler 2012). Also the world population is estimated to double in the next 50 years, so greater yield is required to feed the growing population (Chaves and Davies 2010). The agricultural scenario is thus getting worse as the agricultural land is limited and salt-resistant varieties of crop plants are not available, so there is an emergent need to make the crop plants suitable to this changing scenario.

In this situation, one of the ways is to make the crop plants genetically engineered to sustain their growth and productivity in such challenging environment. For this purpose, it is important to look among the wild relatives of crop plants that carry a good gene pool (Sengupta and Majumder 2009). According to Tester and Bacic (2005), tracing the abiotic stress related genes in the grasses, which are close relatives of cereal crops could lead to the better understanding of the mechanism of salinity tolerance and even introgressing their genes in the cereal genome for inducting better tolerance to salinity. The salt sensitivity of all the grasses is not similar to the cereals and some of these grasses have inherent ability to withstand varying degrees of salt concentrations. According to Flowers et al*.*(2010), the order Poales accounts for approximately 8.1% of the total halophytic species. (Flowers and Colmer 2008) has defined a halophytic species as one that can survive to complete their life cycle in at least 200 mM salt solution. In the last decade the attempt for mining more and more salt tolerant genes from the grasses has increased gradually, also the genes have been characterized and transformed in different model plants to test their efficacy in enhancing salinity tolerance.

In this review, term ‘Salt Tolerant Grasses (STGs)’ has been used for all the grass species including the halophytes, facultative halophytes and salt-tolerant glycophytes. These STGs are a potent source of salt tolerant genes, supported by the occurrence of several traits that works constitutively for imparting salinity tolerance. Thus the STGs offer an ideal system for comparative studies with other cereal crops for mining out more and more novel genes for salt tolerance. Also the non STGs including the cereal crops are also the source of salt tolerant genes, but the prime focus of this review will be on the STGs to understand their salt tolerance mechanism and assess their potentiality as a source of genes for improving the salt tolerance of cereal crops.

### STGs: an overview

The grass family (Poaceae) comprises approximately 10000 species classified in to 600–700 genera (Watson and Dallwitz 1992). According to Gorham et al. (1985) two different mechanisms of salt tolerance are present in plant species growing at high salinities. In succulent Chenopodiaceae, osmotic balance is maintained by the accumulation of Na^+^ and Cl^-^ in shoots followed by synthesis of osmolytes like glycine betaine. In halophytic grasses on the other hand, salt tolerance is mainly achieved either by limiting the influx of salts into the shoots, which results in reduced growth rates under saline conditions or by excluding the excess salt by salt glands. Liphschitz and Waisel (1974) reported 25 species of grasses where salt glands were observed of which mostly were glycophytic, and their distribution was confined to non-saline habitats. Most of the grasses like *Distichlis*, *Aeluropus* of the tribe Aeluropodeae are typically halophytes (Liphschitz and Waisel 1974; Barhoumi et al*.* 2008; Oross and Thomson 1982). Halophytic grasses are highly salt tolerant due to their ability to exclude salt from the internal tissues. Tolerance to salinity in the halophytic grasses like *Puccinellia* and *Thinopyrum* is facilitated by the development of adventitious roots and a superior ability to maintain negative membrane potential in root cells, resulting in greater retention of K^+^ in shoots (Teakle et al. 2013). Also in most of the glycophytic grasses like *Cynodon*, *Sporobolus*, *Zoysia* and *Buchloe* salinity tolerance was positively correlated with Na^+^ and Cl^-^ secretion through salt glands (Marcum 1999). This indicates that the presence of salt glands and Na^+^ exclusion is an important criterion for the primary selection of STGs from the grass species, however the exact mechanism of salinity tolerance in STGs may differ from species to species. Some of the STG species that are discussed in this review for their salt tolerant genes are recorded in Table 1 with their most probable salt tolerance mechanisms that have been focused in different transgenic experiments.

**Table 1:** **STGs and their probable mechanism of action for salt tolerance**

Grass genera	Mechanism of action
*Agropyron*	Compartmentation of ions, Na^+^ ion exclusion
*Aeluropus*	Compartmentation of ions in to vacuoles
*Chloris*	Enhanced ROS scavenging enzymes
*Leymus*	Osmolyte accumulation
*Paspalum*	Osmolyte accumulation
*Pennisetum*	Compartmentation of ions in to vacuoles
*Porteresia*	Osmolyte accumulation
*Puccinellia*	Enhanced ROS scavenging enzymes, Na^+^ ion exclusion
*Spartina*	Compartmentation of ions in to vacuoles

### Mechanisms of salt tolerance in STGs

**Figure 1:**
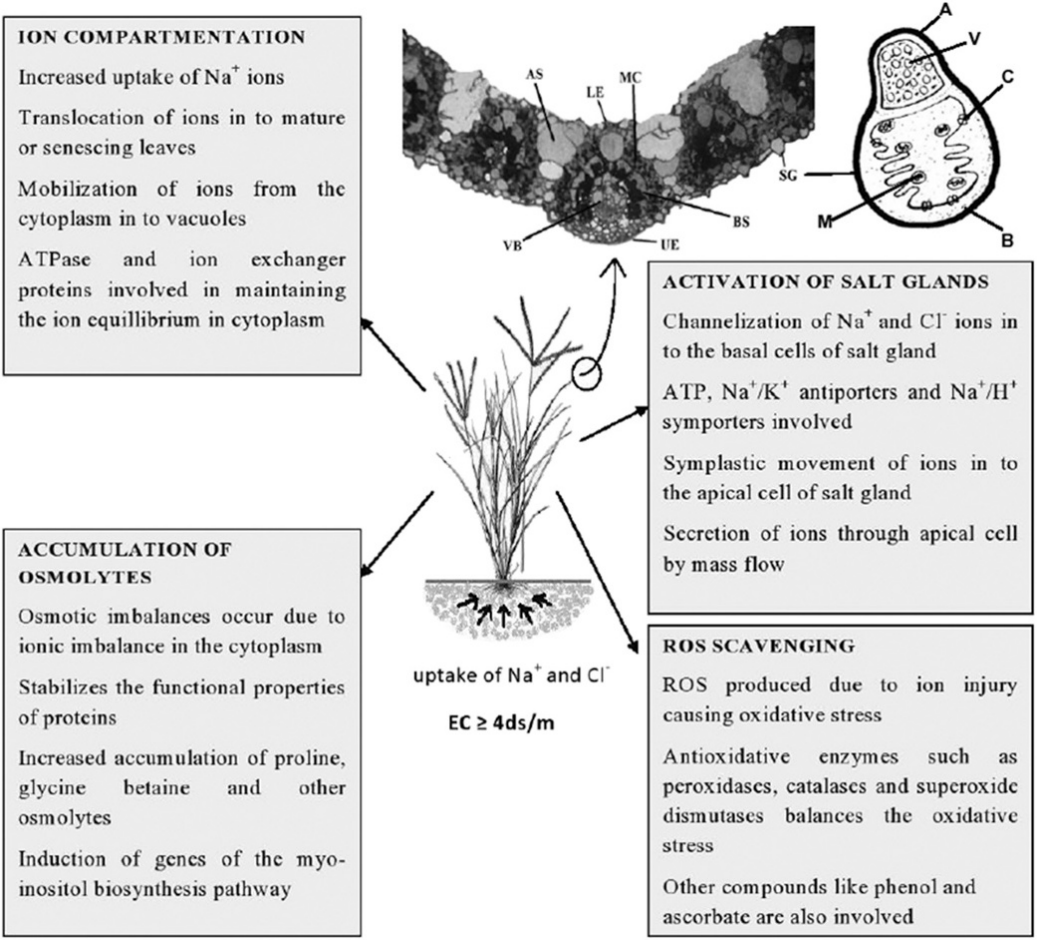
**Generalized scheme showing the different aspects of salinity tolerance in salt tolerant grasses (STGs).** UE- upper epidermis, LE- lower epidermis, BS- bundle sheath, MC- mesophyll cell, VB- vascular bundle, AS- air space beneath stoma, SG- salt gland, A- apical cell, B- Basal cell, M- mitochondria, C– channel proteins, V- vacuole.

The survivability of STGs in saline environment depends greatly up on the art of handling the toxic Na^+^ and replacing it with K^+^. This is known as the selectivity of K^+^ over Na^+^. Net selectivity (S_K: Na_) is defined as the ratio of K^+^ concentration in the plant tissue to that in the medium divided by the ratio of Na^+^ concentration in the plant to that in the medium, which varies between families of flowering plants (Flowers et al. 1986). According to Flowers and Colmer (2008) S_K: Na_ value in flowering plants ranges between average values of 9 and 60 with an overall mean of 19. In the order Poales the S_K: Na_ values of as high as 60 are found. This makes the grasses a strong subject for studying the ion interactions. Marcum (1999) in his studies on the chloridoid grasses and their salinity tolerance mechanisms pointed out that the tolerance was associated with Na^+^ exclusion through the salt glands present on the epidermis of leaves and with accumulation of the compatible solutes like glycine betaine. The overall mechanisms of tolerance in the salt tolerant grasses are summarised in Figure 1.

Tissue-specific compartmentalisation appears to play an important role in most of the grasses (Lessani and Marschner 1978; Taleisnik 1989; Bhatti et al. 1993), where the toxic ions like Na^+^ and Cl^-^ are redistributed to the mature or senescing leaves and other organs. It is widely accepted that cell turgor is maintained by storage of Na^+^ and Cl^-^ in vacuoles, with the solute potential of the cytosol adjusted by accumulation of K^+^ and organic solutes (Storey 1995). Evidence exists for the presence of K^+^/Na^+^ transporter channels across the tonoplast mediated by Na^+^/H^+^ antiport activity, resulting in compartmentation of toxic ions in the vacuoles (Jeschke 1984). Ideally in grasses, Na^+^ and Cl^-^ are largely sequestered in the vacuole of the cell, this sequestering is indicated by the high concentrations of these ions in leaves that are still functioning normally mainly by osmotic adjustments (Marcum 1999).

Salt glands and bladders are the most remarkable organs found in some halophytes which arise from epidermal cells and are modified trichomes (Adams et al*.* 1998). These specialized organs are meant for secretion of toxic ions from the internal part of plant tissues. The salt glands also appear in the STGs that confers increased salinity tolerance in these plants. Salt glands are usually not found in non halophytes (Ramadan and Flowers 2004). Salt glands appear mostly in the mesophyll tissue of C4 grasses. The efficiency of Na^+^ exclusion determines the salt tolerance of a particular species (Marcum 2006). Salt glands are generally bicelled in grasses. In Poaceae, bicellular salt glands are found commonly in the tribes: Chlorideae, Sporoboleae and Aeluropodeae (Liphschitz and Waisel 1974). Salt gland excretion in grasses is highly selective for toxic ions like Na^+^ and Cl^-^ (Worku and Chapman 1998), although other ions like K^+^, Ca^2+^ and Mg^2+^ may also be excreted in small amounts (Marcum and Murdoch 1994). The loading of toxic ions from the adjacent cells in to the salt gland is energy dependent (Naidoo and Naidoo 1999). Flowers et al. (1990) attributed the ability of maintaining a high K:Na ratio in the leaves to the secretion of toxic ions from the leaves. Most of the grass species, which are glycophyte in their habit, can tolerate high levels of salinity due to the presence of salt glands, and also the possession of salt glands suggests that they had a common halophytic ancestor (Liphschitz and Waisel 1974).

Under salinity stress, the osmotic potential of the cytoplasm and organelles is maintained by the accumulation of organic solutes termed as compatible solutes (Flowers et al. 1977; Wyn Jones and Gorham 1983). At lower concentrations, these solutes works by stabilizing the tertiary structure of proteins and enzymes, and function as osmoprotectants (Rhodes et al. 2002). The most likely candidate in the STGs that plays an important role in maintaining the osmotic balance of the plant cells are glycine betaine and proline (Gorham et al. 1986; Rhodes and Hanson 1993). Marcum (1999) in his experiments with Chloridoid grasses found that glycine betaine concentrations under salinity were positively correlated with salinity tolerance in Chloridoid grasses. Lee et al. (2008) reported synthesis of organic compounds in response to salinity stress and their contribution in osmotic adjustments in seashore paspalum grass (*Paspalum vaginatum*). Organic osmolytes that accumulated most importantly in seashore paspalum under salinity stress were proline, glycine betaine, and trigonelline. Marcum and Murdoch (1994) reported that high accumulation of proline in *Cynodon dactylon* kept was associated with the maintenance of osmotic balance, thus providing increased salt tolerance. The accumulation of glycine betaine in salt tolerant grasses like *Cynodon* and *Spartina* were found to be higher than those reported in wheat, sorghum, and other glycophytic grasses (Grieve and Maas 1984; Wyn Jones and Storey 1981). According to Sengupta et al. (2008), inositols like myo-inositol and pinitol also play an important role in stress tolerance mechanism of salt tolerant grasses like *Porteresia coarctata.*

Accumulation of reactive oxygen species (ROS) in plant tissues is highly deteriorating as it can cause oxidative damage to proteins, DNA and lipids (Miller et al*.* 2010). According to Jitesh et al. (2006) most of the halophytes have shown increased efficiency of antioxidative enzymes that helps to negate the harmful effect of ROS. Similarly the STGs also seem to control the Na^+^ induced production of ROS by the increased activity of antioxidative enzymes. Muscolo et al. (2003) studied the activities of different antioxidative enzymes in *Pennisetum clandestinum* in relation to salinity and found that the grass was tolerant to salt stress up to 100 mM NaCl due to the up-regulation of ascorbate peroxidase, peroxidase and glutathione reductase enzymes. Seckin et al. (2010) pointed the role of isozymes of antioxidative enzymes such as peroxidase (POX), catalase (CAT), ascorbate peroxidase (APOX) and superoxide dismutase (SOD) in conferring salt tolerance to *Hordeum marinum* (sea barley grass) plants even at high concentration of NaCl (300 mM), in comparison to *Hordeum vulgare* (cultivated barley).

### Prospect of STGs in cereal crop improvement

#### Model STGs for cereal crop improvement

Rice (*Oryza sativa*) is considered a model plant in monocots because of the relatively small size of its genome, which consists of about 430 Mbp and about 30,000 genes (Komatsu et al. 2003). Apart from rice, *Brachypodium distachyon* has recently emerged as a model system for grass crop genomic research (Hong et al. 2008). But these model plants are glycophytes and naturally lack the ability to adapt and survive in a saline environment, so they are not ideally suited for the dissection of salt stress related genes. Salt tolerance is a complex trait, and therefore requires intensive studies on the tolerance mechanisms of the salt tolerant species of Poaceae (STGs).

**Figure 2:**
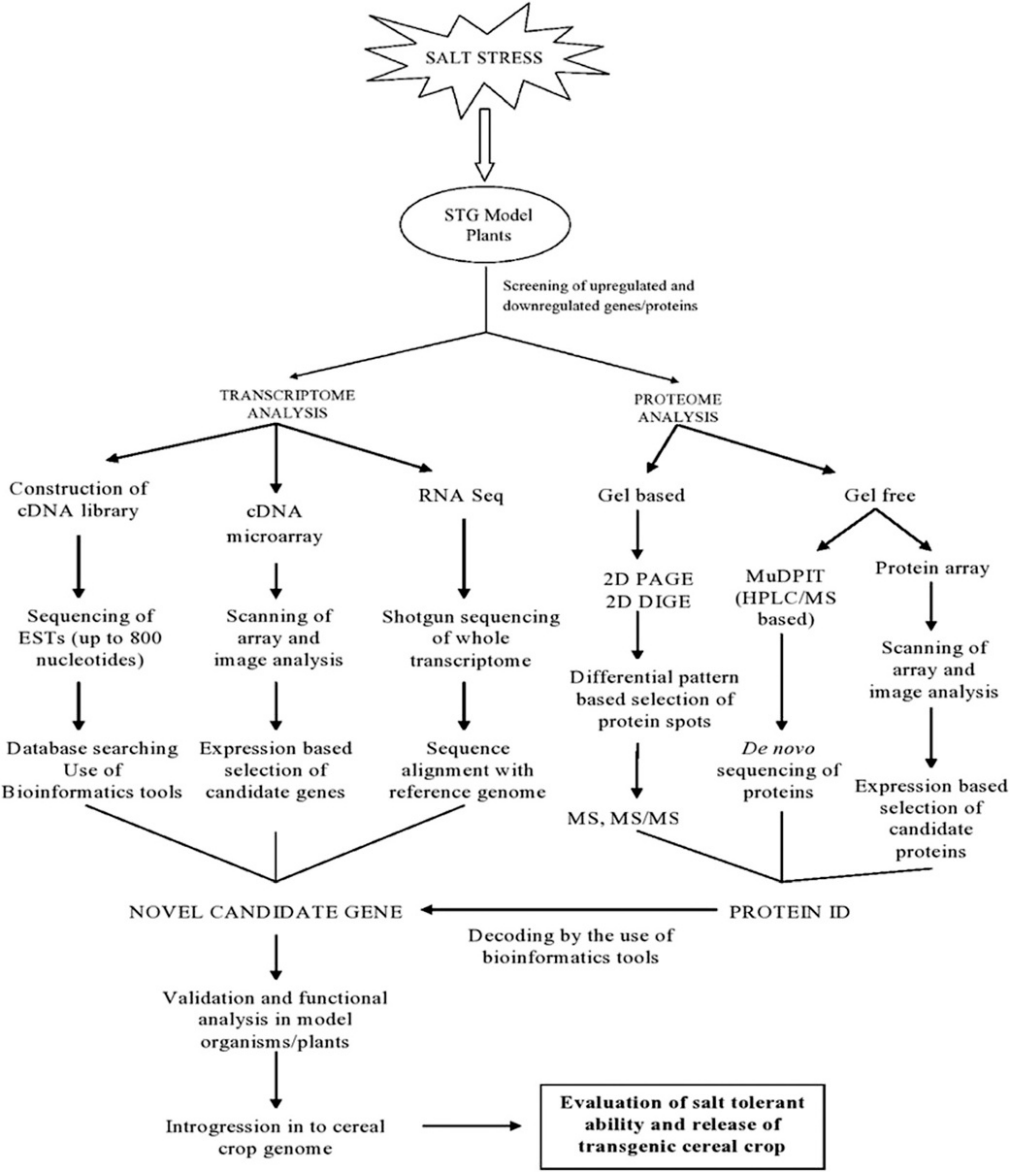
**An outline of tools and techniques through which STG model plants can be employed for cereal crop improvement.**

Sengupta and Majumder (2010) proposed *Porteresia coarctata*, a wild rice, as a potential model for studying salt stress biology in rice. The unique characteristic of this halophytic STG to survive at higher salt concentrations by secretion of excess salts through the leaves has drawn the attention towards it. Due to the availability of complete proteomic information of rice, comparative proteomic study of *Porteresia* in detail would serve the purpose of identification of newer genes and unique salt tolerance mechanisms in the later (Sengupta and Majumder 2009). Also recently Subudhi and Baisakh (2011) proposed *Spartina alterniflora*, as a potent halophytic grass model, as it possesses all known mechanisms of salt tolerance. A comparative analysis of DNA sequences of a full-length myoinositol-1-phosphate synthase gene of *S. alterniflora* (*SaMIPS*) revealed that it is closer to grass species like maize and rice (Baisakh et al. 2008). Other grasses such as *Aeluropus littoralis* and *Puccinellia tenuiflora* also has the potential to become an important source of salt tolerant genes to improve cereal crops (Zouari et al. 2007; Wang et al. 2007). Recent interest on exploring the economically unimportant wild grasses for salt tolerant genes for crop improvement is increasing rapidly and as a result new grasses are being proposed as models for dissection of salt tolerance mechanism and the associated genes. With recent advances, it has become very difficult to concentrate up on a particular species as a model plant. Therefore, it is better to club these potent model plants together, so that the elements of salinity tolerance can be studied together and comparatively. In Figure 2 we have summarized briefly the tools and techniques by which the STG model plants could be employed for cereal crop improvement.

#### Genes conferring salinity tolerance in STGs

A significant progress has been made for the identification of genes and their products, which play an important role in the plant system for overcoming the unfavourable situations of abiotic stresses (Cushman and Bohnert 2000). There are several papers published, that documents intensive works on genetic engineering for abiotic stress tolerance and development of transgenics using different candidate genes (Wu et al. 2005; Roy et al. 2011; Reguera et al. 2012). A number of studies for the identification and characterization of genes have also been done in STGs in response to salinity. Subudhi and Baisakh (2011) reported a number of candidate genes encoding transcription factors, ion transporters, osmoprotectants and antioxidants from salt tolerant *Spartina alterniflora*, upregulated during salt stress. Earlier, analysis of the expression pattern of other genes like cation transport protein (*SaCTP*), vacuolar ATPase (*SaV-ATPase*), and plasma membrane protein 3 (*SaPMP3*) in both leaf and root tissues by reverse transcription PCR (RT-PCR) revealed clear upregulation under salt stress compared to the unstressed control (Baisakh et al. 2008). A number of genes have also been isolated and characterized from *Porteresia coarctata* that are related to the salt tolerance property of the plant, specially the genes operational in inositol metabolic pathway such as myoinositol phosphate synthase (*PcMIPS*) has been analysed in details (Sengupta and Majumder 2009). A putative UDP galactose epimerase gene and a metallothioneine gene was reported to play an important role in conferring salt tolerance to salt tolerant plants of *Paspalum vaginatum* (Endo et al. 2005). Wang et al. (2007) identified 162 unique transcripts corresponding to possible salt-related genes in *P tenuiflora*. Expression of transcripts encoding an aquaporin, V-ATPase subunit B, and the Na^+^/H^+^ antiporter *NHX* was characterized from the salt tolerant *Festuca rubra* ssp. *litoralis* under salt stress (Diedhiou et al*.* 2009). Characterization of salt induced ESTs in salt tolerant *Aeluropus littoralis* revealed that about 20% of the ESTs bear no resemblance in the protein database and are thus novel (Zouari et al. 2007). The analysis of such ESTs could be important in the development of salt tolerant transgenic cereal crops. A significant number of similar studies on other STGs and also in the glycophytic grasses is required to generated a huge database of genes and ESTs that has salt tolerant attributes.

#### Transgenic approaches with genes from STGs

There is an increasing interest to test the novelty of salt tolerant genes through development of transgenic plants. Increased salinity tolerance have been achieved in a range of plant species over expressing *NHX* gene from the halophytes and non-halophytes, which indicates that these genes are involved in Na^+^ tolerance in plants (Guo et al. 2006). Transgenic approaches to salt tolerance are largely achieved through the overexpression of the genes involved in Na^+^ exclusion from the root or leaves and Na^+^ compartmentalization in the vacuoles (Wang et al. 2003; Bhatnagar-Mathur et al. 2008). In the past decade, vacuolar Na^+^/H^+^ antiporters have been the centre of attention in transgenic studies for the alleviation of Na^+^ induced toxicity in plants (Zhang and Blumwald 2001; Vera Estrella et al. 2005; Liu et al. 2008). Several Na^+^/H^+^ antiporter genes have been characterized from STGs and have been showed to impart salt tolerance in transgenic experiments (Qiao et al. 2007; Zhang et al. 2008). Also other genes such as myo-inositol phosphate synthase, ascorbate peroxidase and other transcription factors different STGs and are shown to impart salinity tolerance in transgenic experiments (Majee et al. 2004; Guan et al. 2011; Cheng et al. 2013). Some of the salt tolerant genes derived from STGs that conferred salinity tolerance in different transgenic experiments are listed in Table 2. Some of the salt tolerant genes from non STGs (exclusively grasses) are also mentioned.

**Table 2:** **Genes from STGs and non-STGs imparting enhanced salinity tolerance in transgenic studies**

Source	Gene	Description	Target organism	Inference	Reference
Agropyron elongatum*	*AeNHX1*	Vacuolar Na^+^/H^+^ antiporter	*Arabidopsis* sp.	Root specific compartmentation of Na^+^ ions	Qiao et al. 2007
			*Festuca* sp.		
Aeluropus littoralis*	*AlNHX1*	Vacuolar Na^+^/K^+^ antiporters	*Nicotiana tabacum*	Compartmentalize more Na^+^ in roots and keep a relatively high K^+^/Na^+^ ratio in leaves	Zhang et al. 2008
	*AlSAP*	Two conserved zinc-finger domains A20 and AN1	*Nicotiana tabacum*	Transgenic plants able to set seed at up to 350 mM NaCl	Saad et al. 2010
Chloris virgata*	*ChlMT1*	Metallothioneine	*Saccharomyces*	Increases tolerance to salinity and ROS	Nishiuchi et al. 2007
*Hordeum vulgare*	*HVA1*	Encodes a group of 3 late embryogenesis abundant (LEA) proteins	*Morus alba*	Enhanced salinity tolerance by increased accumulation of proline	Lal et al. 2008
	*HvNHX2*	Vacuolar Na^+^/H^+^ antiporter	*Arabidopsis thaliana*	Able to grow at 200 mM NaCl	Bayat et al. 2011
Leymus chinensis*	*LcDREB3a*	Dehydration-responsive element-binding transcription factor	*Arabidopsis thaliana*	Improved abiotic stress tolerance including salinity with no retardation in growth	Xianjun et al. 2011
	*LcMYB-1*	MYB related transcription factor	*Arabidopsis thaliana*	Enhanced salinity tolerance due to increased accumulation of proline and soluble sugars	Cheng et al. 2013
*Oryza rufipogon*	*OrbHLH001*	Basic helix-loop-helix (bHLH) protein gene	*Arabidopsis thaliana*	Improved salinity tolerance in transgenic plants	Li et al. 2010
*Oryza sativa*	*OsNHX1*	Na^+^/H^+^ antiporter	*Populus* sp.	transgenic plants grew normally in the presence of 200 mM/l NaCl	Wang et al. 2005
Paspalum vaginatum*	*PvUGE1*	Putative UDP-galactose epimerase	*Oryza sativa*	Enhanced salinity tolerance	Endo et al. 2005
	*PvMET1*	Metallothioneine	*Oryza sativa*	Enhanced salinity tolerance	Endo et al. 2005
Pennisetum glaucum*	*PgNHX1*	Vacuolar Na^+^/H^+^ antiporter	*Oryza sativa*	Induced more extensive root system and completion of life cycle of rice at 150 mM NaCl	Verma et al. 2007
Porteresia coarctata*	*PcINO1*	Synthesis of L- myoinositol-1 phosphate synthase	*Nicotiana tabacum*	Tobacco plants showed growth in 200–300 mM NaCl	Majee et al*.*^2004^
	*PcSrp*	Root-specific cDNA encoding serine-rich-protein	*Eleusine coracana*	Transgenic plants able to grow and set seed at 250 mM NaCl	Mahalakshmi et al*.*^2006^
Puccinellia tenuiflora*	*PutAPx*	Ascorbate peroxidase coding gene	*Saccharomyces*	Imparted higher resistance to salinity induced oxidative stress	Guan et al*.*^2011^
	*AKT-1-type-K* ^*+*^ *channel*	Plasma membrane K^+^ channel protein	*Arabidopsis thaliana*	Increased accumulation of K^+^ ion as compared to Na^+^	Ardie et al*.* 2010
Spartina alterniflora*	*SaVHAC1*	Vacuolar H^+^ ATPase subunit C1	*Oryza sativa*	Enhanced salinity tolerance in rice	Baisakh et al*.* 2012
	*SaSce9*	Small ubiquitin related modifier (SUMO) conjugating enzyme	*Arabidopsis thaliana*	Enhanced salinity and drought tolerance by inducing stress responsive genes	Karan and Subudhi 2012
*Triticum aestivum*	*TaNIP*	Nodulin 26-like intrinsic protein (novel aquaporin gene)	*Arabidopsis thaliana*	Enhanced salt tolerance by accumulation of higher K^+^ and proline	Gao et al*.* 2010
	*TaST*	Unknown salt-induced gene	*Arabidopsis thaliana*	Enhanced salt tolerance by accumulating more Ca^2+^, soluble sugar, and proline and less Na^+^	Huang et al*.* 2012
	*TaNHX2*	Vacuolar Na^+^/H^+^ antiporter	*Medicago sativa*	Enhanced salt tolerance	Zhang et al. 2012
*Zea mays*	*ZmPMP3-1*	Plasma membrane protein	*Arabidopsis thaliana*	Enhanced salinity tolerance by regulation of ion homeostasis and ROS scavenging	Fu et al*.* 2012

(*) indicates Grasses considered as STG model plants.

## Conclusion

STGs offer a promising system for the analysis of the mechanisms associated with salinity stress tolerance. The inherent potentiality of these grasses to counteract the negative impacts of salinity is very encouraging for the dissection of more and more novel salt tolerant genes. Also due to their close affinity to the cereal crops, the success of transgenic experiments with the genes from STGs in cereal crops is expected to be very pronounced. Salt tolerance is a complex physiological trait and it has been very difficult to translate the outcome of laboratory experiments in the field, which urges the need for increasing the evaluation of transgenic plants under field conditions (Jewell et al. 2010). Moreover, the very fact that salt tolerance in grasses is an evolutionary labile trait and difficult to transfer in the cereal crops (Bennett et al. 2013), makes it a difficult proposition; still the STGs are emerging as an important plant system that will lead to improved understanding of the gene networks and molecular physiology of cereal crops under salinity stresses. Therefore, further exploration is needed to test the contribution of single or multiple salt stress related genes or regulatory factors from the STGs including *Spartina alterniflora*, *Porteresia coarctata, Puccinellia tenuiflora, Aeluropus littoralis, Agropyron elongatum* and other potent STGs for possible utilization in cereal crop improvement.

## Authors’ original submitted files for images

Below are the links to the authors’ original submitted files for images. Authors’ original file for figure 1 Authors’ original file for figure 2

## Abbreviations

STGs: Salt tolerant grasses
ROS: Reactive oxygen species
POX: Peroxidase
CAT: Catalase
APOX: Ascorbate peroxidase
SOD: Superoxide dismutase
MIPS: Myoinositol phosphate synthase
CTP: Cation transport protein
PMP: Plasma membrane protein
PCR: Polymerase chain reaction
ESTs: Expressed sequence tags.
