# Supplementary figures and images for: Salt tolerance mechanisms in Salt Tolerant Grasses (STGs) and their prospects in cereal crop improvement
Source: Bot Stud. 2014 Mar 14;55:31. doi: 10.1186/1999-3110-55-31 (PMC5432819; doi:10.1186/1999-3110-55-31)

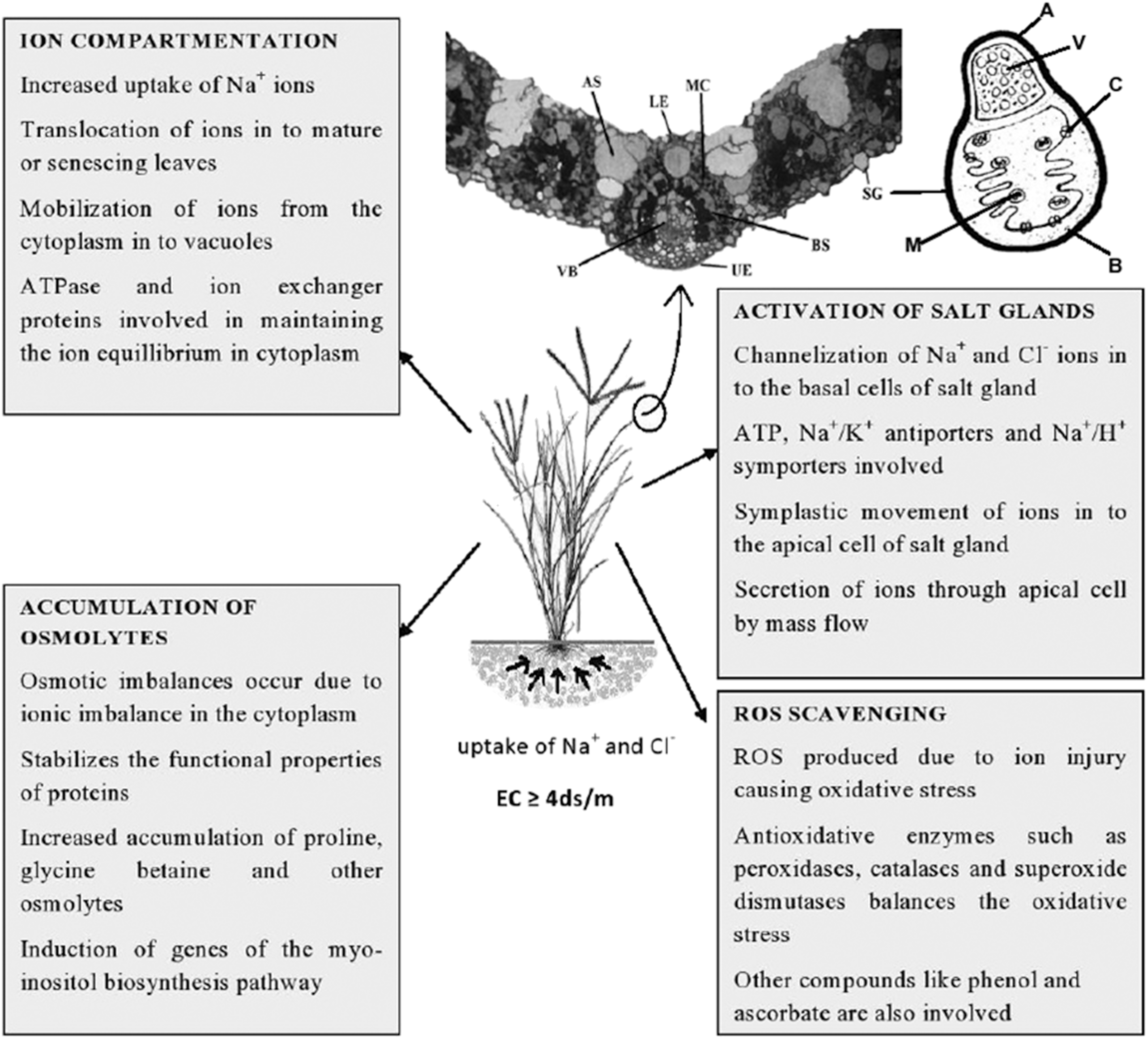

Supplement: Supplementary file 1 — Authors’ original file for figure 1 [file 40529_2014_87_MOESM1_ESM.tiff]

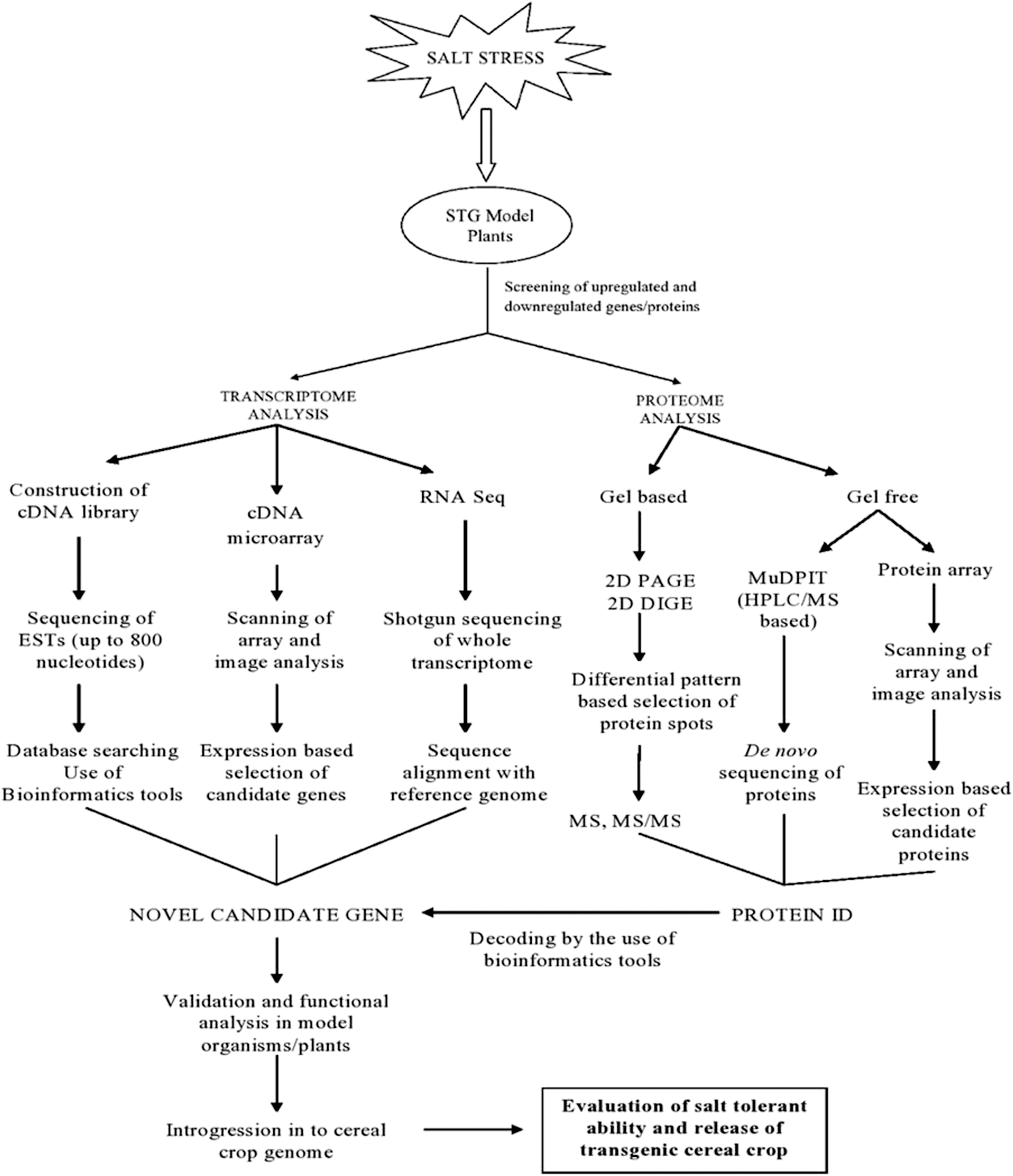

Supplement: Supplementary file 2 — Authors’ original file for figure 2 [file 40529_2014_87_MOESM2_ESM.tiff]
